# Type I interferon score is associated with the severity and poor prognosis in anti-MDA5 antibody-positive dermatomyositis patients

**DOI:** 10.3389/fimmu.2023.1151695

**Published:** 2023-03-17

**Authors:** Jinjing Qian, Rui Li, Zhiwei Chen, Zehui Cao, Liangjing Lu, Qiong Fu

**Affiliations:** ^1^ Department of Rheumatology, Renji Hospital, School of Medicine, Shanghai Jiaotong University, Shanghai, China; ^2^ Institute of Molecular Medicine, Renji Hospital, School of Medicine, Shanghai Jiaotong University, Shanghai, China

**Keywords:** dermatomyositis, anti-MDA5 antibody, interstitial lung disease (ILD), IFN signature, multiplex RT-qPCR assay

## Abstract

**Objectives:**

To investigate the clinical significance of the interferon (IFN) score, especially the IFN-I score, in patients with anti-melanoma differentiation-associated gene 5 (MDA5) antibody-positive dermatomyositis (anti-MDA5^+^ DM).

**Methods:**

We enrolled 262 patients with different autoimmune diseases, including idiopathic inflammatory myopathy, systemic lupus erythematosus, rheumatoid arthritis, adult-onset Still’s disease, and Sjögren’s syndrome, as well as 58 healthy controls. Multiplex quantitative real-time polymerase chain reaction (RT-qPCR) using four TaqMan probes was used to evaluate type I IFN-stimulated genes (IFI44 and MX1), one type II IFN-stimulated gene (IRF1), and one internal control gene (HRPT1), which were used to determine the IFN-I score. The clinical features and disease activity index were compared between the high and low IFN-I score groups in 61 patients with anti-MDA5+ DM. The associations between laboratory findings and the predictive value of the baseline IFN-I score for mortality were analyzed.

**Results:**

The IFN score was significantly higher in patients with anti-MDA5+ DM than in healthy controls. The IFN-I score was positively correlated with the serum IFN-α concentration, ferritin concentration, and Myositis Disease Activity Assessment Visual Analogue Scale (MYOACT) score. Compared with patients with a low IFN-I score, patients with a high IFN-I score showed a higher MYOACT score, C-reactive protein concentration, aspartate transaminase concentration, ferritin concentration, plasma cell percentage, and CD3+ T-cell percentage, as well as lower lymphocyte, natural killer cell, and monocyte counts. The 3-month survival rate was significantly lower in patients with an IFN-I score of >4.9 than in those with an IFN-I score of ≤4.9 (72.9% *vs*. 100%, respectively; P = 0.044).

**Conclusion:**

The IFN score, especially the IFN-I score, measured by multiplex RT-qPCR is a valuable tool to monitor disease activity and predict mortality in patients with anti-MDA5+ DM.

## 1 Introduction

Inflammatory myopathies are a group of systemic autoimmune diseases that affect the muscles, skin, and other organs (1, 2). Anti-melanoma differentiation-associated gene 5 (MDA5) antibody is highly associated with a specific type of inflammatory myopathy known referred to as anti-MDA5+ dermatomyositis (anti-MDA5+ DM). MDA5, which is also named interferon induced with helicase C domain 1 (IFIH1), is a cytoplasmic sensor of viral RNA that activates a cascade of antiviral responses, including type I interferon (IFN) signaling and other proinflammatory cytokines (3, 4). Patients with anti-MDA5 antibody positivity are prone to mild or no muscle involvement and typical cutaneous manifestations, and they frequently develop rapidly progressive interstitial lung disease (RP-ILD) (5, 6). Of note, patients with RP-ILD usually do not respond to conventional immunosuppressive treatment. However, Janus kinase (JAK) inhibitors combined with glucocorticoids significantly improve the survival of patients with early-stage anti-MDA5+ DM with interstitial lung disease (7). However, the 6-month mortality rate remains high, especially in East Asian regions (5, 7).

The involvement of type 1 IFN in the pathogenesis of anti-MDA5+ DM has been proposed based on significant elevation in the expression of downstream stimulated genes in muscle, skin, lung, and peripheral blood. Several studies have demonstrated the presence of an elevated type I IFN signature in muscle biopsies from patients with DM, including anti-MDA5+ DM (8, 9). Furthermore, sarcoplasmic MxA expression detected by immunohistochemistry is a sensitive marker for diagnosing DM, and it reflects the severity of muscle involvement in patients with juvenile DM (10–12). Similarities to muscle disease also exist in the skin. For example, the skin lesions of patients with DM show type I IFN-induced recruitment of CXCR3+ lymphocytes, and robust expression of the type I IFN marker MxA has been demonstrated in both blood vessels and interstitial fibroblasts in the skin of patients with anti-MDA5+ DM (13–15). In the lungs of patients with anti-MDA5+ DM, increased proportions of interferon-stimulated gene ISG+ CD4+ T cells and ISG+ CD8+ T cells were detected, further highlighting overactivation of the type I IFN pathway in the affected lungs of patients with anti-MDA5+ DM (16). Compared with other cytokine-induced genes, such as the genes encoding tumor necrosis factor-α (TNF-α), interleukin (IL)-10, and IL-1β, the type I IFN signature in the blood is positively correlated with disease activity in individual patients with dermatomyositis (DM) or polymyositis (PM) during longitudinal follow-up according to a previous study (17). These findings suggest that the type I IFN signature might serve as a useful biomarker and could be used as a tool to monitor clinical efficacy outcomes, which is useful because the clinical management of patients with anti-MDA5+ DM is challenging.

Poor sensitivity and specificity have made enzyme-linked immunosorbent assay measurements of circulating IFN challenging (18). Despite the fact that RNA sequencing can yield a vast amount of data, it is time-consuming, which is not optimal in clinical practice where timely feedback is required to monitor disease progression. To set up a clinical routine test to measure the IFN score quickly and easily, we established a multiplex RT-qPCR assay to simultaneously measure four genes in a single reaction, which allows the two IFN families to be differentiated, including two type I IFN-stimulated genes, one type II IFN-stimulated gene, and one internal control gene.

By applying multiplex RT-qPCR, we assessed type I IFN activity by examining gene expression in peripheral blood mononuclear cells (PBMCs) obtained from patients with anti-MDA5+ DM to determine whether the IFN score could be used to differentiate anti-MDA5+ DM from other autoimmune diseases and different myopathy subtypes. Additionally, we examined whether the IFN score, especially the type I IFN score, correlates with disease activity in patients with anti-MDA5+ DM, along with its association with laboratory findings and ability to predict 3-month mortality.

## 2 Materials and methods

### 2.1 Patients, clinical features, and laboratory data

A total of 262 patients, including 61 patients with anti-MDA5^+^ DM and 201 patients with other autoimmune diseases, were recruited from February 2021 to September 2022 at the Department of Rheumatology, Renji Hospital, Shanghai, China. The study protocol was approved by the Ethics Committee of Renji Hospital (ID: 2013-126), Shanghai, China. Informed consent was obtained from the study participants. This study was conducted according to the Declaration of Helsinki. Patients with anti-MDA5^+^ DM fulfilled the modified Sontheimer’s criteria (19, 20) and were screened for myositis-specific autoantibodies (MSAs) and myositis-associated antibodies. Blood samples were collected at baseline, and the data were retrospectively obtained, including demographic information, laboratory data, clinical features, and measurements of idiopathic inflammatory myopathy (IIM) disease activity based on the Myositis Disease Activity Assessment Visual Analogue Scale (MYOACT) and The Cutaneous Dermatomyositis Disease Area and Severity Index (CDASI). Fifty-eight healthy volunteers with no known diseases were included as controls.

### 2.2 Detection of myositis-specific autoantibody

MSAs, including anti-MDA5 antibody, were analyzed using a commercially available immunoblot assay (EUROLINE Autoimmune Inflammatory Myopathies 16 Ag (IgG); Euroimmun, Lubeck, Germany).

### 2.3 PBMC isolation and RNA extraction

Whole blood was collected in ethylenediaminetetraacetic acid tubes (BD, 367863) and stored at 4°C until PBMC isolation. Serum samples were collected simultaneously. Human PBMCs were isolated using Lymphoprep (Stemcell, 07801), and erythrocytes were lysed with red blood cell lysis buffer (Tiangen, RT122-02). Following the manufacturer’s instructions, RNA was extracted from PBMCs using Direct-zol RNA Miniprep Kits (Zymo, R2052) and was used as a template for quantitative real-time polymerase chain reaction (RT-qPCR).

### 2.4 Real-time PCR analysis and scoring data

Multiplex Taqman-based RT-qPCR was performed to determine the expression of a panel of genes. HPRT1 was used as a housekeeping gene/internal control. The target genes were IFI44, Mx1/MxA, and IRF1. The relative expression of each target gene (T/R) was calculated using the 2^−ΔCT^ method. The T/R of each target gene was then normalized as follows: (T/R_subject_ − mean_HC_) ÷ SD_HC_, where HC is healthy control and SD is standard deviation. The IFN score was calculated as the mean of the normalized T/R of the three target genes. Scores higher than the mean of the HC plus two SDs were designated as high IFN scores; otherwise, the IFN score was considered low.

### 2.5 Statistical analysis

The Mann–Whitney U test with Bonferroni correction was performed to identify differences in variables between the high IFN-I score group and the low IFN-I score group. The chi-square test or Fisher’s exact test was used to compare categorical variables, as appropriate. Correlations between laboratory findings and disease activity indices were assessed using Spearman’s Rho. All graphs depict the mean ± SD, unless otherwise stated. All analyses were performed using SPSS (version 26.0), and a two-tailed P value of <0.05 was considered statistically significant.

## 3 Results

### 3.1 Multiplex RT-qPCR based on three IFN-stimulated genes revealed IFN pathway activation in autoimmune diseases

**Figure 1:**
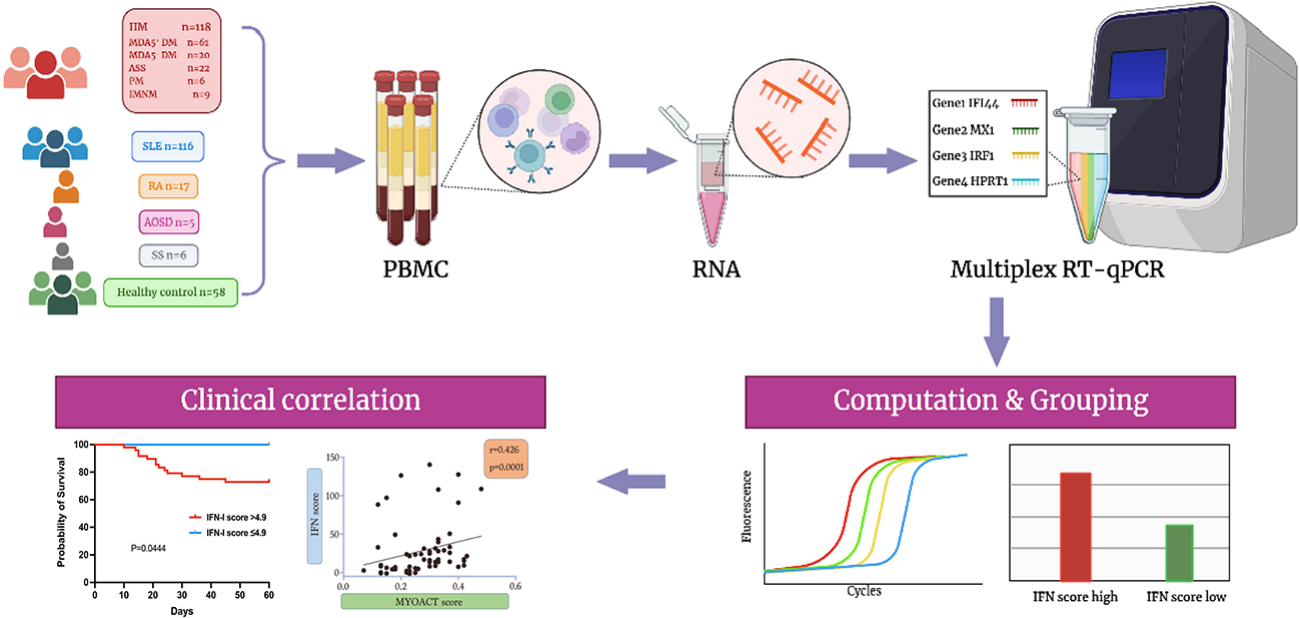
Flowchart illustrating the overall experimental design of the study. Biorender and Adobe Illustrator were used to create the image.

We collected peripheral blood samples from patients with five distinct autoimmune diseases, as well as from healthy controls. Among the 118 patients diagnosed with IIM, 61 had anti-MDA5+ DM, 20 had anti-MDA5^−^ DM, 22 had anti-synthetase syndrome (ASS), six had polymyositis (PM), and nine had IMNM (immune-mediated necrotizing myopathy). There were 116 patients with systemic lupus erythematosus (SLE), 17 with rheumatoid arthritis (RA), five with adult-onset Still’s disease (AOSD), six with Sjögren’s syndrome (SS), and 58 healthy controls. Using RNA isolated from PBMCs as the template, multiplex RT-qPCR was performed to simultaneously quantify the relative expression of four genes, including two type I IFN-stimulated genes (*IFI44* and *MX1*), one type II IFN-stimulated gene (*IRF1*), and one housekeeping gene (*HPRT1*) (**Figure 1**). After dividing the relative expression of each target gene by the mean of the normalized expression in HCs, the IFN score was calculated as the median of the relative expression of all contributing genes. The mean plus two SDs of the HC values was set as the cutoff for identifying a high IFN score. Therefore, patients with anti-MDA5+ DM were divided into two groups (the high and low IFN score groups). As shown in **Figure 1**, a significant clinical association was found between IFN-I score and MYOACT score.

### 3.2 IFN score in PBMC distinguishes anti-MDA5+DM from disease controls and health controls

**Figure 2:**
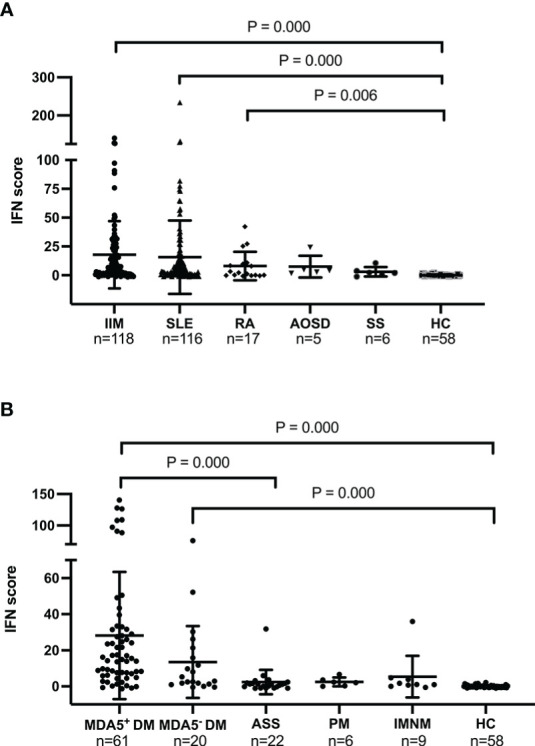
Baseline IFN scores in different autoimmune diseases and in healthy controls. **(A)**. IFN scores in the IIM, SLE, RA, AOSD, SS, and HC groups. **(B)**. IFN scores in the anti-MDA5+ DM, anti-MDA5− DM, ASS, PM, INIM, and HC groups. The Kruskal–Wallis test with Bonferroni correction was performed using SPSS software (version 23.0). The IFN score of each individual is represented as a single dot. Horizontal lines represent the mean ± SD. IFN, interferon; IIM, idiopathic inflammatory myopathy; SLE, systemic lupus erythematosus; RA, rheumatoid arthritis; AOSD, adult-onset Still’s disease; SS, Sjögren’s syndrome; HC, healthy control.

We compared the IFN scores among patients with different autoimmune diseases. **Figure 2A** shows that patients with IIM (P = 0.000), SLE (P = 0.000), and RA (P = 0.006) had significantly higher IFN scores than healthy controls. No significant differences were found between patients with AOSD or SS and patients in the other groups. Remarkably, IIM and SLE were the two types of autoimmune disease with the most highly upregulated IFN scores. As stated previously, anti-MDA5+ DM is a distinct subtype of IIM with a high mortality rate. Therefore, we compared the IFN scores of various subtypes of IIM with the IFN score of HCs. Anti-MDA5+ DM had the highest IFN score of all IIM subtypes. Patients with anti-MDA5+ DM had significantly higher IFN scores than patients with ASS (P = 0.000) and HCs (P = 0.000) (**Figure 2B**). Patients with anti-MDA5− DM also had a significantly higher IFN score than HCs (P = 0.000). Based on the analysis of the 20 patients of MDA5-DM, the study found that 15% (3 out of 20) exhibited anti-SAE1 antibodies. In addition, 30% (6 out of 20) of patients had anti-NXP2 antibodies, 30% of patients had anti-TIF1-γ antibodies,15% (3 out of 20) had anti-Mi-2 antibodies, and 10% (2 out of 20) had anti-Ro52 antibodies. No significant differences were found between the IFN scores of the ASS, PM, IMNM, and HC groups.

### 3.3 Characteristics of patients with anti-MDA5+DM

IFN-I has been recognized as a key player in the pathogenesis of anti-MDA5 + DM, with several studies highlighting its involvement in the disease. Therefore we decided to focus primarily on the IFN-I score in this study. The baseline characteristics of patients with anti-MDA5+ DM are listed in **Table 1**. On the basis of the IFN-I score, 51 of 61 patients with anti-MDA5+ DM were assigned to the high IFN-I score group, while 10 patients were assigned to the low IFN-I score group. A statistically significant difference in disease duration was found at 9.68 ± 13.36 months in the high IFN-I score group and at 49.80 ± 13.70 months in the low IFN-I score group (P = 0.007). In terms of age, gender, clinical features, and treatments, there were no significant differences between the high and low IFN-I score groups. There were 6 patients with anti-MDA5 antibodies who were diagnosed with classic DM and 56 patients with anti-MDA5 antibodies who were diagnosed with clinically amyopathic dermatomyositis (CADM).

**Table 1:** Baseline characteristics of patients with anti-MDA5+ DM and comparisons between the high and low IFN-I score groups.

	Overall (n = 61)	IFN-I high (n = 51)	IFN-I low (n = 10)	p-value
Demographic				
Age (year)	53.46 ± 12.85	54.18 ± 12.69	49.80 ± 13.70	0.391
Gender (female, %)	67.21	68.63	60.00	1
Disease duration (month)	12.04 ± 18.83	9.68 ± 13.36	49.80 ± 13.70	0.007*
IFN-I score	28.18 ± 35.28	33.58 ± 36.22	0.61 ± 1.46	0.000*
Clinical manifestations				
Heliotrope rash (%)	22 (36.10)	19 (37.30)	3 (30.00)	0.735
Gottron sign (%)	41 (67.20)	36 (70.60)	5 (50.00)	0.273
Skin ulceration (%)	9 (14.80)	7 (13.70)	2 (20.00)	0.633
Periungual erythema (%)	21 (34.40)	20 (39.20)	1 (10.00)	0.143
Arthritis (%)	12 (19.70)	12 (23.50)	0 (0.00)	0.187
Shawl-sign (%)	5 (8.20)	5 (9.80)	0 (0.00)	0.580
V-sign (%)	4 (6.56)	4 (7.84)	0 (0.00)	0.421
Mechanic’s hands (%)	4 (6.56)	4 (7.84)	0 (0.00)	0.421
Treatment				
Dose of GC (mg)	54.46 ± 50.30	60.09 ± 52.84	30.25 ± 28.05	0.097
GC (%) CsA (%)	52 (98.11) 1 (1.60)	42 (97.7) 1 (2.00)	10 (100.0) 0 (0.00)	0.8111
MMF (%)	3 (4.90)	2 (3.90)	1 (10.00)	0.421
TAC (%)	22 (36.10)	18 (35.30)	4 (40.00)	1
CTX (%)	7 (11.50)	5 (9.80)	2 (20.00)	0.322
JAKi(%) IVIG (%)	15 (24.60) 6 (10.00)	12 (23.50) 6 (11.8)	3 (30.00) 0 (0.00)	0.696 0.587

Categorical variables were compared using the chi-square test or Fisher’s exact test, as appropriate, while continuous variables were compared using the Student’s t-test or the Mann–Whitney U test. GC, glucocorticoid; CsA, cyclosporine A; TAC, tacrolimus; MMF, mycophenolate mofetil; CTX, cyclophosphamide; JAKi, JAK inhibitor; IVIG, intravenous immunoglobulin. *P < 0.05.

### 3.4 The IFN-I score is related to the clinical characteristics of patients with anti-MDA5^+^ DM

**Figure 3:**
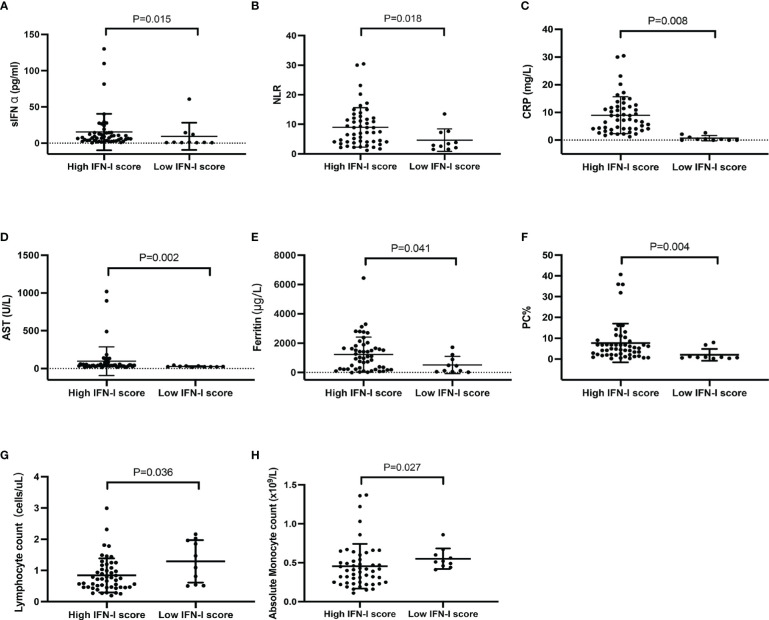
Comparison of laboratory data between patients with anti-MDA5+ DM in the high (n = 51) and low (n = 10) IFN-I score groups. **(A)** Serum IFN-α concentration, **(B)** NLR, **(C)** CRP concentration, **(D)** AST concentration, **(E)** ferritin concentration, **(F)** PC%, **(G)** lymphocyte count, and **(H)** absolute monocyte count. Comparisons were performed using the Mann–Whitney U test. The bars represent the mean ± SD. NLR, neutrophil-to-lymphocyte ratio; CRP, C‐reactive protein; AST, aspartate transaminase; PC, plasma cell.

The Mann–Whitney U test was performed to understand whether the laboratory test results differed between the high and low IFN-I score groups. The serum IFN-α concentration (15.34 ± 25.19 pg/ml *vs*. 9.32 ± 18.75 pg/ml, P = 0.015), neutrophil-to-lymphocyte ratio (8.96 ± 6.67 *vs*. 4.63 ± 3.78, P = 0.018), C-reactive protein (CRP) concentration (4.30 ± 6.65 mg/L vs. 0.67 ± 0.94 mg/L, P = 0.008), aspartate transaminase (AST) concentration (96.75 ± 189.70 U/L vs. 27.00 ± 7.86 U/L, P = 0.002), and ferritin concentration (1225.30 ±1192.26 μg/L vs. 514.28 ± 584.03 μg/L, P = 0.041), as well as the percentage of peripheral plasma cells (7.74% ± 9.30% *vs*. 2.03% ± 2.85%, P = 0.004) and CD3+ T cells (65.12% ± 12.15% *vs*. 53.16% ± 19.00%, P = 0.039), were significantly higher in the high IFN-I score group than in the low IFN-I score group (**Figures 3A–F**, **Figure S1C**). In contrast, patients with anti-MDA5+ DM in the high IFN-I score group had lower lymphocyte (0.83 ± 0.54 cells/μL vs. 1.29 ± 0.68 cells/μL , P = 0.036) and absolute monocyte (0.45 ± 0.29 x109/L vs. 0.55 ± 0.13 x109/L, P = 0.027) counts than patients in the low IFN-I score group (**Figures 3G, H**). The natural killer cell count (92.77 ± 100.22 cells/μL vs. 353.91 ± 437.46 cells/μL, P =0.004) and the percentage of CD3− CD16+ CD56+ natural killer cells (11.59% ± 8.21% *vs*. 26.29% ± 21.09%, P = 0.029) were also lower in the high IFN-I score group (**Figures S1A–B**).

### 3.5 Correlation of the IFN-I score with laboratory findings and disease activity in patients with anti-MDA5^+^DM

**Figure 4:**
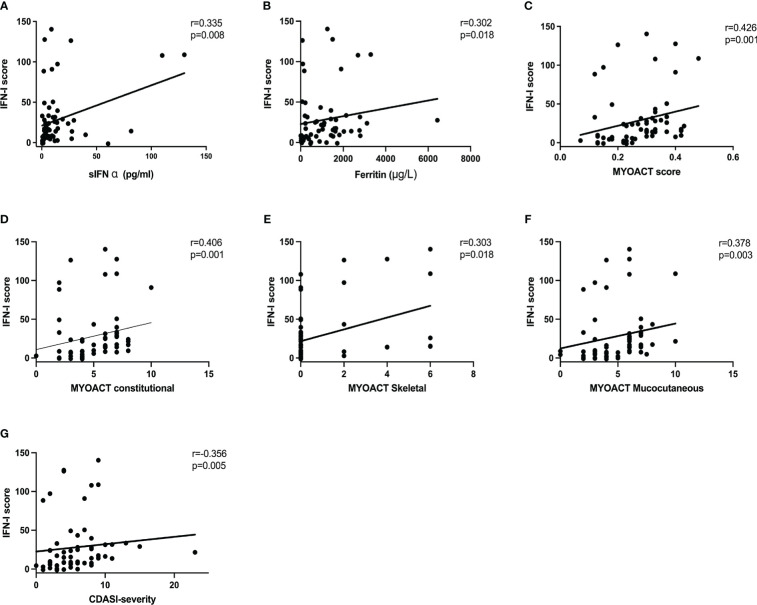
Correlation of the IFN-I score with disease activity parameters and clinical features. The IFN-I score in patients with anti-MDA5+ DM is associated with the concentrations of serum IFN-α **(A)** and ferritin **(B)**. The IFN-I score also correlated with the MYOACT score **(C)**, MYOACT constitutional **(D)**, MYOACT skeletal **(E)**, MYOACT mucocutaneous **(F)**, and severity based on the CDASI **(G)**. The data were analyzed using Spearman’s Rho.CDASI, cutaneous dermatomyositis disease area and severity index; MYOACT, Myositis Disease Activity Assessment Visual Analogue Scale.

We examined the correlations of the IFN-I score with the laboratory findings and disease activity in patients with anti-MDA5+ DM. Moderately positive correlations between the IFN-I score and serum IFN-α concentration (r = 0.335, P = 0.008), ferritin concentration (r = 0.302, P = 0.018), AST concentration (r = 0.343, P = 0.007), and percentage of peripheral plasma cells (r = 0.362, P = 0.004) were observed (**Figures 4A, B**, **Figures S2A–B**). In contrast, the IFN-I score was negatively correlated with the absolute natural killer cell count (r = −.0360, P = 0.004), the percentage of CD3-CD16+CD56+ natural killer cell (r = −.0364, P = 0.004), and the disease duration (r = −.0317, P = 0.013) (**Figure S2C–E**). The results show a positive association between the IFN-I score and laboratory findings. Overall, the IFN-I score was positively correlated with the MYOACT score (r = 0.426, P = 0.001) (**Figure 4C**). In terms of individual organ systems, the IFN-I score was moderately positively correlated with the MYOACT constitutional (r = 0.406, P = 0.001), MYOACT skeletal (r = 0.303, P = 0.018), MYOACT mucocutaneous (r = 0.378, P = 0.003), and severity according to the CDASI (r = 0.356, P = 0.005) (**Figures 4D–G**). These results imply that the IFN-I score is positively correlated with myositis disease activity in patients with anti-MDA5+ DM.

### 3.6 A high IFN-I score identifies patients with active MDA5+DM and predicts mortality

**Figure 5:**
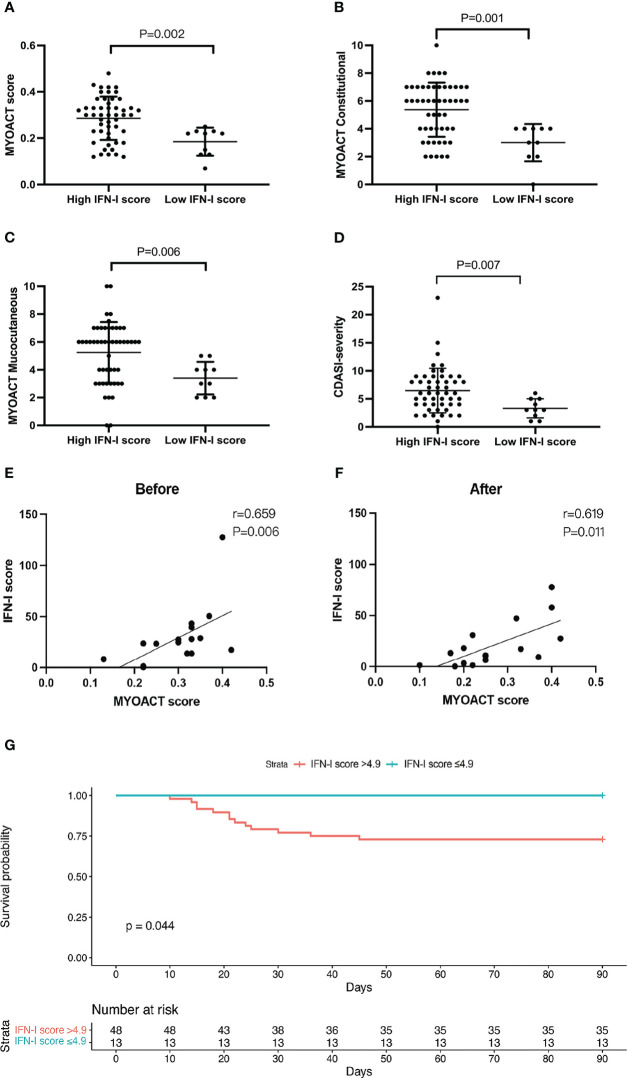
A high IFN-I score identifies patients with active anti-MDA5+ DM and predicts mortality. Grouped analysis of MYOACT scores and CDASI scores at baseline **(A–F)**, **(A)** MYOACT score, **(B)** MYOACT constitutional, **(C)** MYOACT mucocutaneous, and **(D)** severity based on the CDASI. Patients were divided into two groups based on the IFN-I score (high *vs*. low). Comparisons were performed using the Mann–Whitney U test. **(E, F)** Correlations of the IFN-I score and the MYOACT score in 16 patients before and after treatment. **(G)** The Kaplan–Meier survival curve shows the survival rates stratified according to the IFN-I score. The high IFN-I score group had an IFN-I score of >4.9 (n = 48), whereas the low IFN-I score group had an IFN-I score of ≤4.9 (n = 13). Comparisons were performed using the log-rank test.

We examined the difference in disease activity index between the high and low IFN-I score groups. Compared with the low IFN-I score group, the high IFN-I score group had a significantly higher MYOACT score (P=0.006), MYOACT constitutional (P = 0.001), MYOACT mucocutaneous (P = 0.001), and severity based on the CDASI (P = 0.007) (**Figures 5A–D**). In contrast, MYOACT skeletal, MYOACT pulmonary, and CDASI damage were unaffected (**Figures S3A–C**).

We analyzed the change in the IFN-I score in 16 patients during hospitalization. The results show that the IFN-I score paralleled the MYOACT disease activity index before (**Figure 5E**) and after (**Figure 5F**) treatment. During the observation period, 13 of 61 patients died of respiratory failure induced by interstitial lung disease. A significant difference was observed in the 3-month survival rate of patients with an IFN-I score of >4.9 and those with an IFN-I score of ≤4.9 (72.9% *vs*. 100%, respectively; P = 0.044) (**Figure 5G**). This suggests that a high IFN-I score of >4.9 may serve as a sensitive marker to predict mortality in patients with anti-MDA5+ DM.

## 4 Discussion

DM is a heterogeneous group of autoimmune diseases that can be subdivided based on the presence of various MSAs. Patients with anti-MDA5+ DM exhibit typical cutaneous manifestations and interstitial lung disease, but myositis is either less severe or non-existent in these patients. The poor prognosis results from the fact that traditional immunosuppressive treatment is frequently ineffective in patients with anti-MDA5+ DM with interstitial lung disease, especially rapidly progressive interstitial lung disease, which is a significant factor contributing to mortality. From the therapeutic perspective, additional simple and sensitive biomarkers should be identified to evaluate disease activity and forecast the disease course of patients with anti-MDA5+ DM. The selection of IFI44, Mx1, and IRF1 as targeted genes in this study was based on several considerations, including their previous identification as highly upregulated and specific interferon-stimulated genes (21–23), the need to differentiate between IFN-I and IFN-II signaling pathways, and the importance of selecting genes with close expression and designing non-mutually exclusive primers for accurate and reliable detection using multiplex RT-qPCR assays. The application of multiplex RT-qPCR in this study of IFN-I scores in patients with anti-MDA5+ DM was innovative. The limitation of not being able to differentiate between the two IFN families was overcome by this approach, which covers both type I and type II IFN-stimulated genes, whereas previous gene sets only allowed differentiation into high and low IFN scores (24). As anticipated, the IFN-I score was increased in more than 80% of patients with anti-MDA5+ DM and was significantly higher in individuals with anti-MDA5+ DM than in patients with SLE, RA, AOSD, and SS. Meanwhile, 23% of patients with anti-MDA5+ DM exhibited elevated IFN-II scores and differences in CRP concentration, ALT concentration, and MYOACT skeletal were observed between the high IFN-II score and the low IFN-II score groups (**Figure S4**). Previous research has suggested that increased expression of IFN-II may contribute to the development and severity of MDA5+DM (25). However, the number of significant variations associated with IFN-II score was fewer than expected. This may be because IFN-II has a local effect on the tissue microenvironment and could not be easily detected in blood circulation. Thus, we primarily focused on analyzing the IFN-I score, as it appeared to be more strongly associated with practical parameters than the IFN-II score. The IFN-I score reflected the disease severity because it was significantly associated with ferritin concentration, AST concentration, and MYOACT scores. During the course of hospitalization, the IFN-I score was associated with disease activity. Notably, anti-MDA5 antibody-positive patients with an IFN-I score of >4.9 were likely to have a worse prognosis than those with an IFN-I score of ≤4.9, which may aid in the clinical care of these patients. Thus, patients with an IFN-I score of >4.9 may respond to anti-type I IFN therapy, such as JAK inhibitors. These results imply that the IFN-I score may contribute to the pathogenesis of anti-MDA5+ DM and could potentially serve as a diagnostic tool for this disease. However, it’s important to note that further research is needed to fully understand the role of IFN-II in this disease, as our findings highlight the importance of both IFN-I and IFN-II in anti-MDA5+ DM.

The results of this study were in accordance with those of a previous study by Higgs et al., who showed that the type I IFN signature can be found in the blood of patients with SLE, RA, PM, Systemic Sclerosis (SSc), and DM (26). Compared with ASS and other myopathies, DM is typically associated with a strong type I IFN signature (27, 28). These characteristics were corroborated in the present study by the existence of the IFN-I signature in the blood of patients with anti-MDA5+ DM.

Earlier investigators compared the IFN scores between disease types. In the current investigation, in addition to evaluating the IFN score between selected diseases, patients with anti-MDA5+ DM were separated into two groups (the high and low IFN-I score groups). Intriguingly, the percentage of plasma cells in the high IFN-I score group was considerably higher than in the low IFN-I score group. This result may be because RNA-containing immune complexes generated by MDA5 and anti-MDA5 are potent inducers of IFN-α through Toll-like receptor-7, and type I IFN promotes B-cell differentiation into plasma cells (29–32). In a multivariate logistic regression model developed by Xu et al., anti-MDA5 positivity, a CRP concentration of >50 μg/L, and a lymphocyte count of <500/μL predicted rapidly progressive interstitial lung disease with an overall accuracy of 90% (33). In a recent study, the lymphocyte and monocyte counts of the non-survivor group in the first month were significantly lower than the survivor group (34). In this study, the high IFN-I score group also had a higher CRP concentration and decreased lymphocyte and monocyte counts. These findings suggest that in clinical practice, patients with a reasonably high IFN-I score, either alone or in combination with other serum indicators, should be prioritized for higher mortality.

The IFN-I score has been used in the past to quantify disease activity. The type I IFN signature score in the blood is strongly associated with disease activity in DM and PM during baseline evaluation and longitudinal follow-up according to previous studies (17, 26, 27, 35). The disease activity index, as well as the ferritin, serum IFN-α, and AST concentrations, was positively correlated with the IFN-I score in the present study. IFN-α promotes the synthesis of ferritin (36). Horai et al. discovered that anti-MDA5+ individuals with interstitial lung disease had higher IFN-α and ferritin concentrations than anti-MDA5− patients with interstitial lung disease (37). According to Lian et al. and Gono et al., the serum ferritin concentration is significantly higher in patients with anti-MDA5+ DM and predicts the disease severity and prognosis of rapidly progressive interstitial lung disease (38–40). Ferritin was also elevated in the high IFN-I score group, indicating that it could be useful to monitor disease activity and assist in stratifying high-risk groups. A statistically significant difference in disease duration was found between high and low IFN-I score groups suggesting treatment over a longer period could lead to decreasing levels of biomarkers. The lack of significant differences in the use of GC, the dosage of GC, and several immunosuppressants between high and low IFN-I groups as shown in **Table 1** suggest that medication use may not be a major confounding factor in the cross-sectional study analysis of the IFN-I score. It is possible that low IFN-I score patients have milder conditions and therefore longer survival time, which could contribute to a longer follow-up period while patients with a shorter disease duration may have a more acute and severe form of the disease. However, long duration of treatment and high doses of GCs and/or immunosuppressants might affect the levels of cytokines and gene expression, which can in turn influence the levels of IFN-I scores. Exploring and studying the use of a normal or decreasing IFN-I score as a target to be achieved with drug therapy could be a promising area of research to improve the treatment of anti-MDA5-positive dermatomyositis.

Multiple markers are associated with the prognosis of anti-MDA5+ DM, including anti-MDA5 titers (41, 42), the presence of anti-Ro52 antibody (43), lactate dehydrogenase (18), ferritin (5, 24, 26), KL-6 (27), the proportion of CD4+ CXCR4+ T cells (28), and a high proportion of ISG15+ CD8+ T cells (16). The presence of interstitial lung disease, particularly rapidly progressive interstitial lung disease, poses significant obstacles to the prognosis of patients with anti-MDA5+ DM. Overactivation of the type I IFN pathway is apparent in the affected lungs of patients with anti-MDA5+ DM according to a previous study (16). Type I IFN recruits CX3CR1+ M2 macrophages in the lungs by inducing the secretion of CX3CL (44, 45), and M2 macrophages are responsible for producing tumor growth factor-β to promote pulmonary fibrosis (46–48). However, little previous evidence supports the idea that the IFN score in patients with anti-MDA5+ DM is directly associated with a poor prognosis. We propose that an IFN-I score of >4.9 could be useful to predict outcomes and assess treatment efficacy, especially in patients treated with targeted synthetic disease-modifying antirheumatic drugs, thereby addressing a previously unfulfilled need. The lack of a strong association between the IFN-I score and the MYOACT pulmonary score in patients with MDA5+ DM suggests that the IFN-I score may not be a reliable indicator of lung involvement in these patients. It is possible that the IFN-I score reflects a more systemic immune response rather than a localized response in the lungs. However, A high IFN-I score is still considered an indicator of a high-risk immune and inflammatory state that is linked to disease severity and poor prognosis. Therefore, patients with a high IFN-I score should still receive clinical attention and aggressive treatment, as this may be an early warning sign before the onset of significant pulmonary symptoms.

This study had several limitations. First, anti-MDA5+ DM is relatively uncommon, and thus the research was limited by the small sample size. Second, the study was performed at a single medical center in Shanghai, China. Therefore, external validation and large-scale studies are required to validate the findings. Third, pulmonary function testing was not performed because the overall medical condition of some patients was pretty severe, and these patients could therefore not undergo pulmonary function testing. Finally, due to their severe medical condition, some patients were already being treated with high glucocorticoid doses or JAK inhibitors at the time of admission, which is likely to interfere with the type I IFN pathway and can cause alterations in gene expression. Hence, large-scale studies in patients with the new-onset disease are required.

## 5 Conclusion

The present study offers a novel approach to comprehending the dysregulated type I IFN production in patients with anti-MDA5+ DM and understanding how it relates to the disease severity. In the future, dissecting the cellular and molecular mechanisms underpinning this process will be essential. Precise risk assessment based on clinical phenotypes and IFN scores to help implement early targeted and personalized therapy in high-risk patients may significantly improve the long-term prognosis of patients with anti-MDA5+ DM.

## Equations


$$Normalized\;{T/R}_{subject}=({T/R}_{subject}-{mean}_{HC})÷{SD}_{HC}$$

## Data availability statement

The original contributions presented in the study are included in the article/**Supplementary Material**. Further inquiries can be directed to the corresponding authors.

## Ethics statement

The studies involving human participants were reviewed and approved by The Ethics Committee of Renji Hospital (ID: 2013-126), Shanghai, China. The patients/participants provided their written informed consent to participate in this study.

## Author contributions

QF, LL and ZHC supervised the study and revised the manuscript. JQ performed the experiments and drafted the manuscript. RL and ZWC collected and analyzed clinical data. All authors contributed to the article and approved the submitted version.
