# Supplementary material for: Type I interferon score is associated with the severity and poor prognosis in anti-MDA5 antibody-positive dermatomyositis patients
Source: Front Immunol. 2023 Mar 17;14:1151695. doi: 10.3389/fimmu.2023.1151695 (PMC10063972; doi:10.3389/fimmu.2023.1151695)
Supplement: Supplementary file 1 [file DataSheet_1.docx]

Supplementary Material

Type I interferon score is associated with the severity and poor prognosis in anti-MDA5 antibody-positive dermatomyositis patients

Jinjing Qian, MD1†, Rui Li, MD 1 †, Zhiwei Chen, MD 1 , Hanlin Yin, MD 1 , Zehui Cao, PhD2*, Qiong Fu, PhD1*, Liangjing Lu, PhD1*

*** Correspondence:**

Liangjing Lu: [lu_liangjing@163.com](mailto:lu_liangjing@163.com)

Qiong Fu: [fuqiong5@163.com](mailto:fuqiong5@163.com)

Zehui Cao: zincimm@outlook.com

# Supplementary Data

Supplementary Material should be uploaded separately on submission. Please include any supplementary data, figures and/or tables.

Supplementary material is not typeset so please ensure that all information is clearly presented, the appropriate caption is included in the file and not in the manuscript, and that the style conforms to the rest of the article.

# Supplementary Figures and Tables

For more information on Supplementary Material and for details on the different file types accepted, please see [here](https://www.frontiersin.org/guidelines/author-guidelines#supplementary-material).

## Supplementary Figures
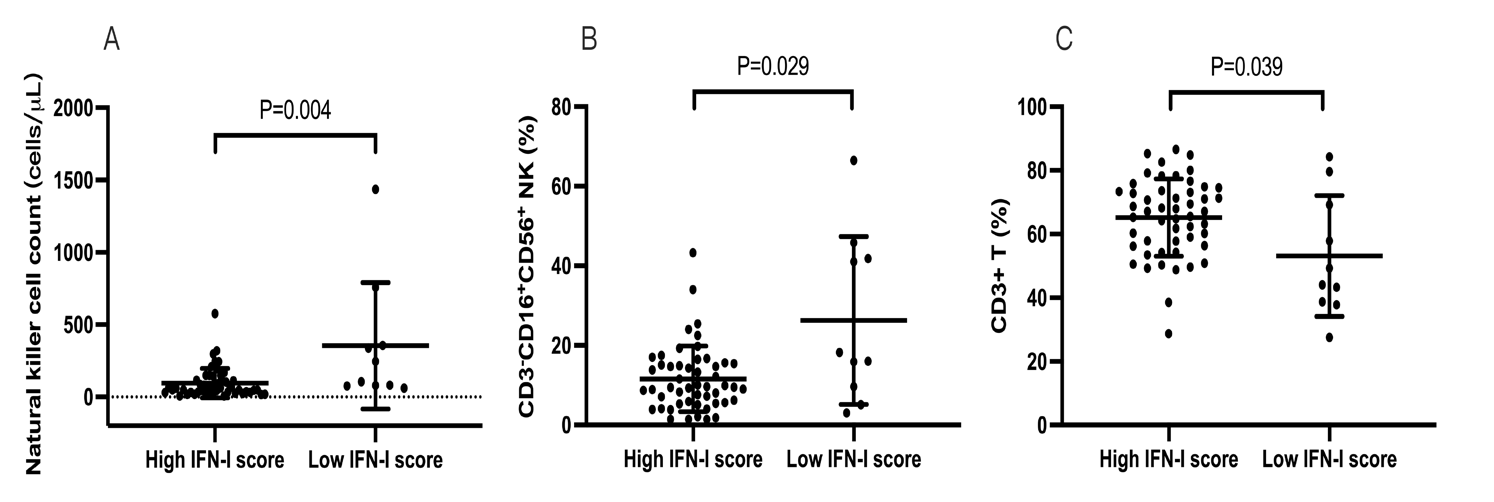


## Supplementary Figure 1. Comparison of lymphocyte subsets between patients with anti-MDA5+ DM with high and low IFN-I scores.

## (A) Natural killer cell count, (B) percentage of CD3−CD16+CD56+ natural killer cells, and (C) percentage of CD3+ T cells. DM, dermatomyositis; IFN-I, interferon-I; NK, natural killer cell.

**
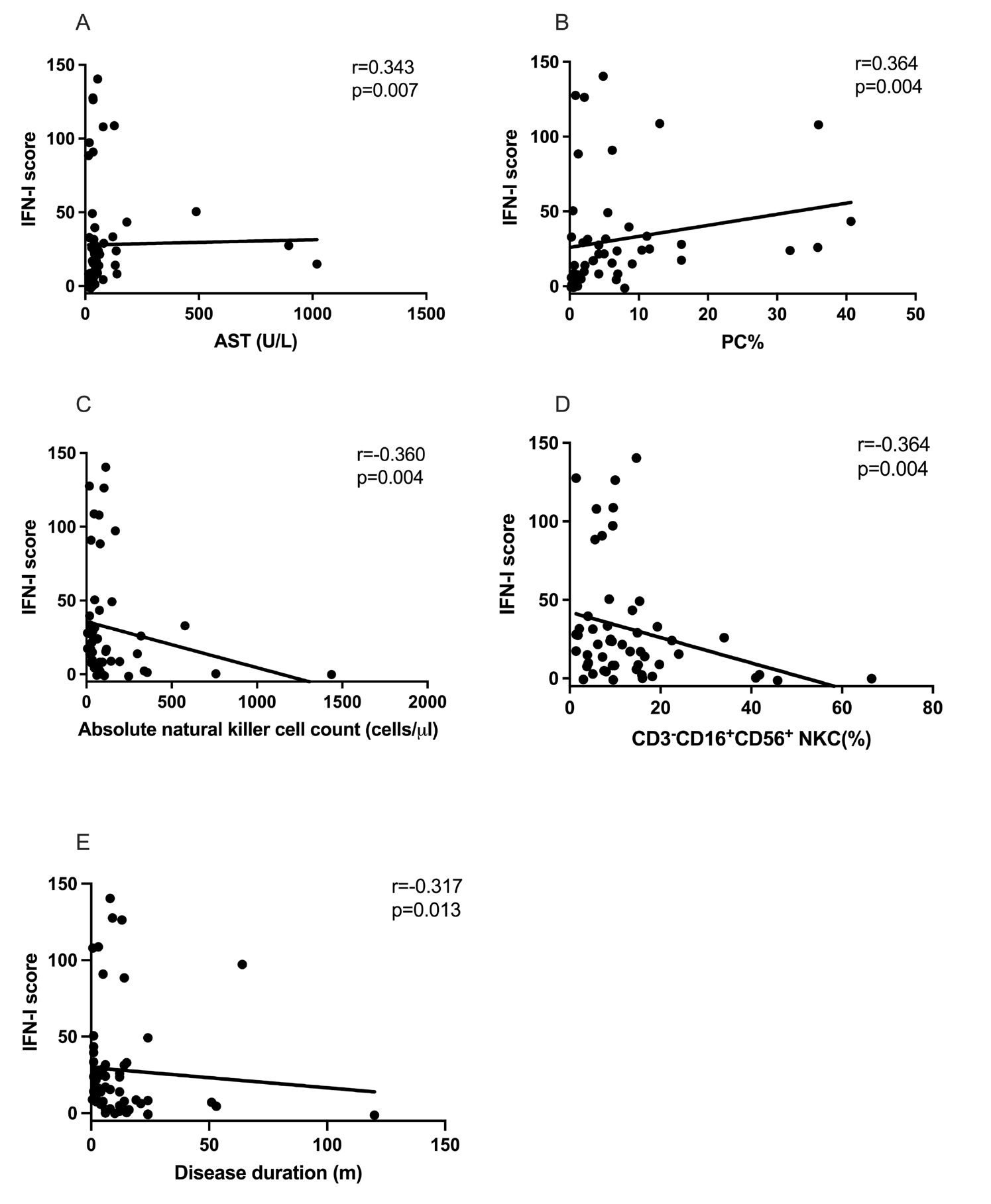
Supplementary Figure 2.** Correlations between the IFN-I score and clinical features.

The IFN-I score correlated with (A) AST concentration, (B) percentage of plasma cells, (C) absolute natural killer cell count, (D) percentage of CD3− CD16+ CD56+ natural killer cells, (E) and disease duration. IFN, interferon; AST, aspartate aminotransferase; PC%, percentage of plasma cells; NKC, natural killer cell


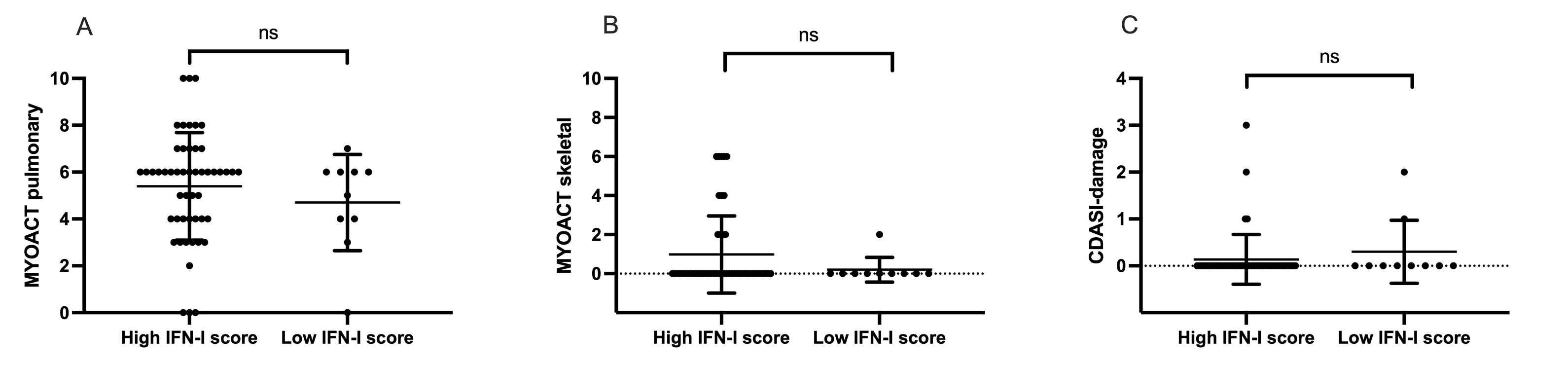


**Supplementary Figure 3.** Comparisons of MYOACT indices between patients with anti-MDA5+ DM with high and low IFN-I scores

(A) MYOACT pulmonary, (B) MYOACT skeletal, and (C) damage based on the CDASI.

IFN, interferon; CDASI, cutaneous dermatomyositis disease area and severity index; MYOACT, Myositis Disease Activity Assessment Visual Analogue Scale; DM, dermatomyositis


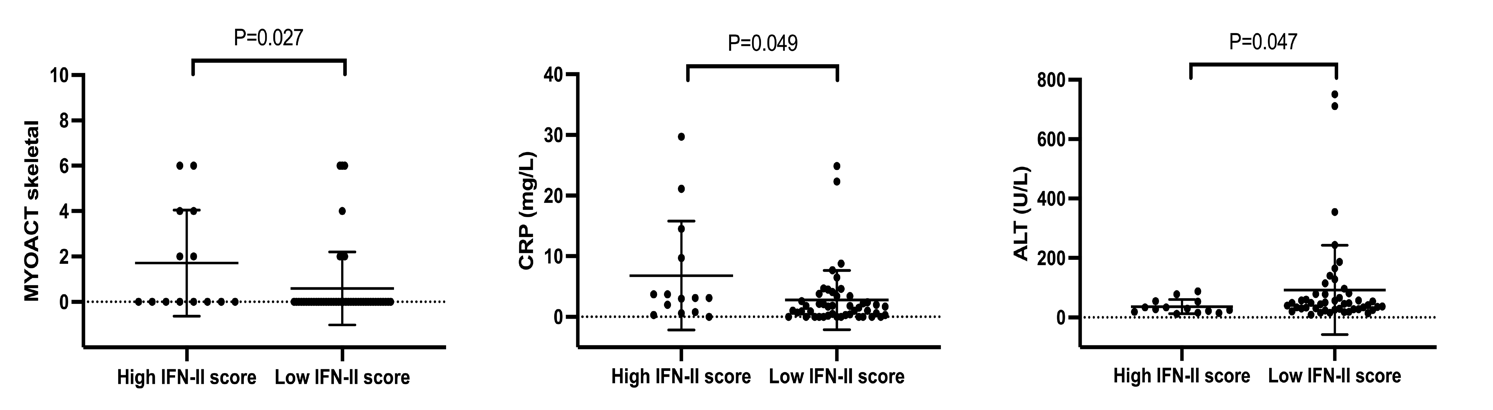


**Supplementary Figure 4.** Comparisons between patients with anti-MDA5+ DM with high and low IFN-II scores

(A) MYOACT skeletal, (B) CRP concentration, and (C) ALT concentration. IFN, interferon; CRP, C‐reactive protein; ALT, alanine aminotransferase; MYOACT, Myositis Disease Activity Assessment Visual Analogue Scale; DM, dermatomyositis
